# Comparison of Serratus Posterior Superior Intercostal Plane Block and Pectoral Nerve Block II for Postoperative Analgesia Following Modified Radical Mastectomy: A Retrospective Cohort Study

**DOI:** 10.3390/jcm15218757

**Published:** 2026-11-09

**Authors:** Hilal Hande Başaran, Canan Salman Önemli, Derya Arslan Yurtlu, Pelin Uzun Sarıtaş, Zeki Tuncel Tekgül, Cem Karaali

**Affiliations:** 1 Department of Anesthesiology and Reanimation, İzmir City Hospital, 35540 Izmir, Türkiye; canan_ege_35@hotmail.com (C.S.Ö.); darslanyurtlu@yahoo.com (D.A.Y.); puzun77@hotmail.com (P.U.S.); cem.karaali@sbu.edu.tr (C.K.); 2 Department of Anesthesiology and Reanimation, İzmir Faculty of Medicine, University of Health Sciences, 35540 Izmir, Türkiye; 3 Department of Anesthesiology and Reanimation, Izmir Bazekol Çiğli Hospital, 35630 Izmir, Türkiye; zekittekgul@yahoo.com; 4 Department of General Surgery, İzmir Faculty of Medicine, University of Health Sciences, 35540 Izmir, Türkiye

**Keywords:** modified radical mastectomy, postoperative analgesia, regional anesthesia, breast neoplasms, serratus posterior superior intercostal plane block, PECS II block

## Abstract

**Background/Objectives**: To compare the postoperative analgesic efficacy of the serratus posterior superior intercostal plane (SPSIP) block and pectoral nerve block II (PECS II) in patients undergoing unilateral modified radical mastectomy (MRM). **Methods**: This single-center retrospective cohort study included 100 patients who underwent unilateral MRM and received either an SPSIP block (*n* = 34) or a PECS II block (*n* = 66). The primary outcome was the time from extubation to the first administration of rescue analgesia. Secondary outcomes included Numeric Rating Scale (NRS) pain scores at 1, 4, 8, and 12 h postoperatively, the maximum NRS pain score, and cumulative opioid consumption within the first 24 postoperative hours. The primary outcome was analyzed using a generalized linear model with a gamma distribution and log-link function adjusted for operative duration. A sensitivity analysis additionally adjusted for age, body mass index, American Society of Anesthesiologists (ASA) physical status, and smoking status. **Results**: Among 100 patients (SPSIP, *n* = 34; PECS II, *n* = 66), operative duration was longer in the PECS II group (*p* < 0.001). Time to first rescue analgesia was longer with SPSIP (7.5 [5.0–8.8] vs. 5.0 [3.0–8.0] h; *p* = 0.004), but the difference was not significant after adjustment for operative duration (exp(B) = 1.21, 95% CI 0.98–1.49; *p* = 0.077) or additional covariates (exp(B) = 1.17, 95% CI 0.94–1.46; *p* = 0.153). NRS scores were lower with SPSIP at 1 h (*p* < 0.001) and with PECS II at 8 and 12 h (Holm-adjusted *p* = 0.013 and *p* < 0.001). Twenty-four-hour tramadol consumption was similar (*p* = 0.922). No block-related complications were documented. **Conclusions**: After adjustment for operative duration, the longer time to first rescue analgesia observed in the SPSIP group did not retain statistical significance, and a similar result was obtained in the sensitivity analysis. The findings should be interpreted cautiously in view of the retrospective design and limited sample size.

## 1. Introduction

Modified radical mastectomy (MRM) remains one of the most commonly performed surgical procedures for the treatment of breast cancer. Despite advances in surgical techniques and perioperative care, acute postoperative pain continues to represent a major clinical challenge because of extensive surgical dissection involving both the breast and axillary regions. Inadequately controlled postoperative pain may delay recovery, impair early mobilization, increase opioid requirements and opioid-related adverse effects, and contribute to the development of persistent postsurgical pain. Consequently, effective perioperative analgesia has become an essential component of multimodal analgesic strategies and enhanced recovery pathways following breast surgery.

The pectoral nerve block II (PECS II) is one of the most extensively investigated fascial plane blocks for breast surgery, targeting the innervation of the pectoral region. The pectoral nerves originate from the brachial plexus and innervate the pectoral muscles. The lateral pectoral nerve arises from C5, C6, and C7, whereas the medial pectoral nerve originates from C8 and T1. The lateral pectoral nerve courses between the pectoralis major (PM) and pectoralis minor muscles and innervates the PM muscle [1].

The other group of nerves comprises the anterior divisions of the thoracic intercostal nerves from T2 to T6. These nerves course through the intercostal spaces, giving off lateral and anterior branches as they proceed toward the sternum [1].

In the PECS II block, the first local anesthetic injection targets the lateral and medial pectoral nerves, whereas the second injection, administered between the pectoralis minor and serratus anterior muscles, targets the lateral branches of the intercostal nerves (T3–T6) and the intercostobrachial nerve [1].

Randomized controlled trials and meta-analyses have demonstrated that PECS II effectively reduces postoperative pain intensity and opioid consumption following breast surgery [2,3,4,5]. However, the duration and magnitude of its analgesic effect may vary in more extensive procedures, such as MRM with axillary lymph node dissection.

The serratus posterior superior intercostal plane (SPSIP) block is a recently described fascial plane technique developed for thoracic analgesia [6]. The serratus posterior superior muscle, which attaches to the lateral margins of the second through fifth ribs, extends between the C7 and T2 vertebral levels. Given its anatomical location at the cervical and upper thoracic vertebral levels, the muscle receives its innervation primarily from the ventral rami of the upper intercostal nerves (T2–T5) and the lower cervical nerves. This anatomical arrangement enables the SPSIP block to provide sensory blockade across dermatomal regions encompassing the back, neck, shoulder, axilla, and lateral chest wall, extending cephalically to C3 and caudally to T7 [6].

The SPSIP block has been reported to provide analgesia in various settings, including persistent myofascial pain, breast surgery, thoracic surgery, and shoulder surgery.

Although the initial evidence supporting the use of this technique in breast surgery was limited to case reports, subsequent randomized controlled trials have suggested that the SPSIP block may provide postoperative analgesia in this patient population [7,8,9,10,11]. Nevertheless, clinical evidence directly comparing SPSIP with PECS II remains limited.

In the SPSIP block, local anesthetic is injected into the fascial plane between the serratus posterior superior muscle and the second and third ribs. Compared with the local anesthetic injections administered into the fascial plane between the pectoralis major and pectoralis minor muscles and between the pectoralis minor and serratus anterior muscles in the PECS II block, this injection is performed in a fascial plane farther from the surgical site. We hypothesized that potential anatomical disruption at the injection planes used for the PECS II block, resulting from their proximity to the surgical field, as well as possible damage to vascular structures, could affect local anesthetic spread and the onset of analgesic action. Accordingly, this study aimed to compare the postoperative analgesic efficacy of the SPSIP block and the PECS II block in patients undergoing unilateral MRM. To this end, the duration of analgesia, postoperative pain intensity, and postoperative analgesic requirements were evaluated.

## 2. Materials and Methods

### 2.1. Study Design and Ethical Approval

This retrospective single-center cohort study was conducted by reviewing the perioperative medical records of consecutive patients who underwent unilateral modified radical mastectomy (MRM) between December 2023 and December 2024. The study protocol was approved by the İzmir City Hospital Non-Interventional Clinical Research Ethics Committee (Approval No. 2025/24; 22 January 2025) and was conducted in accordance with the ethical principles of the Declaration of Helsinki. Owing to the retrospective design of the study, the requirement for written informed consent was waived by the Ethics Committee.

### 2.2. Study Population

Female patients aged 18–65 years with an American Society of Anesthesiologists (ASA) physical status of I–III who underwent unilateral MRM and received either an ultrasound-guided serratus posterior superior intercostal plane (SPSIP) block or pectoral nerve block II (PECS II) for postoperative analgesia were eligible for inclusion.

The sample size was determined by the number of eligible patients who underwent unilateral modified radical mastectomy and received either an SPSIP or a PECS II block during the study period.

Patients who underwent bilateral breast surgery, simultaneous breast reconstruction, or had incomplete perioperative or postoperative analgesia records precluding assessment of the study outcomes were excluded.

### 2.3. Anesthetic Management

Institutional anesthesia records demonstrated that all patients received standardized intraoperative monitoring, including electrocardiography, non-invasive blood pressure measurement, pulse oximetry, and bispectral index (BIS) monitoring.

General anesthesia was induced with intravenous lidocaine (1 mg/kg), propofol (2–3 mg/kg), fentanyl (1 μg/kg), and rocuronium (0.6 mg/kg), followed by endotracheal intubation. Anesthesia was maintained with sevoflurane and a continuous remifentanil infusion.

The drugs and doses administered were in accordance with our institution’s standard general anesthesia protocol. All patients received fentanyl (1 μg/kg) during induction, and remifentanil was administered as a continuous intraoperative infusion. Because cumulative remifentanil dose and individual infusion requirements were not consistently available in the retrospective records, actual intraoperative remifentanil exposure could not be compared between the groups.

As part of the institutional multimodal analgesia protocol, all patients received intravenous paracetamol (1000 mg) and tramadol (50 mg) approximately 30 min before extubation.

No longer-acting opioids or other analgesic drugs were administered before emergence from anesthesia or at the end of surgery.

### 2.4. Regional Block Techniques

Both SPSIP and PECS II blocks were performed under ultrasound (GE LOGIQ P9) guidance by anesthesiologists experienced in ultrasound-guided regional anesthesia, after completion of the surgical procedure and before extubation, concurrently with the administration of intravenous analgesics.

Operating room personnel assignments were determined according to the daily surgical schedule, with no fixed pairing between individual surgeons and attending anesthesiologists. Consequently, the composition of the surgical and anesthesia teams varied across cases. The regional analgesic technique was selected by the attending anesthesiologist on the basis of routine clinical practice and individual clinical judgment, rather than according to a predefined allocation protocol.

The SPSIP block was performed as follows: The ultrasound probe was placed transversely at the level of the scapular spine to visualize the superomedial border of the scapula, the trapezius muscle, the rhomboid major (Rm) muscle, the serratus posterior superior muscle (SPSM), and the second and third ribs. The probe was then rotated 90 degrees into a parasagittal orientation from the posterior aspect of the supraclavicular fossa to identify the first rib. The second and third ribs were subsequently confirmed. The probe was positioned to obtain an inferomedial-to-superolateral view of the superomedial border of the scapula. The needle was advanced immediately medial to the scapula, targeting the region between the second and third ribs to reach the fascial plane between the SPSM and the intercostal muscles. A total of 30 mL of 0.25% bupivacaine was injected into the fascial plane between the serratus posterior superior muscle and the second and third ribs.

The PECS II block was performed using the conventional two-injection technique described by Blanco, consisting of a PECS I injection between the pectoralis major and pectoralis minor muscles followed by a second injection between the pectoralis minor and serratus anterior muscles [1].

The PECS II block was performed as follows: Initially, the ultrasound probe was positioned longitudinally over the clavicle at the midclavicular line, posterior to the first rib. The thoracoacromial artery was visualized by moving the probe inferiorly to the third intercostal space. At this level, 10 mL of 0.25% bupivacaine was injected between the pectoralis major and pectoralis minor muscles (PECS I block). This injection blocks the medial and lateral pectoral nerves. The probe was then rotated 45° toward the axilla. By moving the probe toward the anterior axillary line, the attachment of the serratus anterior muscle immediately superficial to the rib was visualized. At this level, 20 mL of 0.25% bupivacaine was injected between the pectoralis minor and serratus anterior muscles at the third intercostal space.

### 2.5. Postoperative Analgesia and Outcome Measures

Following surgery, intravenous paracetamol was administered routinely every 8 h. Pain intensity was assessed using the Numeric Rating Scale (NRS) at 1, 4, 8, and 12 h after extubation.

In our clinic, rescue analgesia is standardized according to postoperative pain intensity in patients receiving regional blocks. Provided that the NRS score is ≥4, rescue analgesia is consistently administered to all patients as intravenous tramadol (50 mg).

At each postoperative pain assessment, the NRS score was recorded before the administration of rescue analgesia. Rescue analgesic administrations and their timing were documented in the medical records. Therefore, NRS scores obtained at later postoperative time points were interpreted in relation to any rescue analgesia administered previously.

The primary outcome was the time from extubation to the first administration of rescue analgesia.

Secondary outcomes included postoperative NRS pain scores at predefined time points, maximum postoperative NRS score, and cumulative tramadol consumption during the first 24 postoperative hours. The tramadol dose administered before extubation was not included in the cumulative postoperative opioid consumption.

### 2.6. Statistical Analysis

Statistical analyses were performed using IBM SPSS Statistics for Windows (Version 22.0; IBM Corp., Armonk, NY, USA), with additional effect-size and bootstrap analyses performed using Python (Python Software Foundation, https://www.python.org/).

Continuous variables were assessed for normality using the Shapiro–Wilk test, histogram inspection, and normal probability plots. Continuous variables are presented as the median and interquartile range (IQR), whereas categorical variables are presented as frequencies and percentages.

Between-group comparisons of continuous variables were performed using the Mann–Whitney U test. Categorical variables were analyzed using Pearson’s chi-square test or Fisher’s exact test, as appropriate.

For secondary outcomes analyzed using the Mann–Whitney U test, rank-biserial correlation (rᵣᵦ) was calculated as an effect-size measure, with corresponding 95% confidence intervals (CIs) estimated using 5000 bootstrap resamples. Effect-size estimates were expressed for SPSIP relative to PECS II; negative values indicate lower values in the SPSIP group, whereas positive values indicate higher values in the SPSIP group.

For between-group comparisons of NRS scores at the four predefined postoperative time points (1, 4, 8, and 12 h), the Holm procedure was applied to adjust for multiple comparisons.

To evaluate the association between regional block type and the primary outcome, a generalized linear model (GENLIN) with a gamma distribution and log-link function was constructed. Because the primary outcome consisted exclusively of positive, right-skewed values and the time to first rescue analgesia was completely observed for all included patients, this modeling approach was considered appropriate. No censoring, truncation, or imputation was applied to the primary outcome.

The primary adjusted model included block type and operative duration. To assess the robustness of the findings, a sensitivity analysis was additionally performed after adjustment for age, body mass index (BMI), ASA physical status, and smoking status.

Model fit was assessed using scaled deviance and Pearson chi-square goodness-of-fit statistics. Exponentiated regression coefficients [exp(B)] are reported with corresponding 95% CIs and represent ratios of model-based expected mean times to first rescue analgesia; they should not be interpreted as hazard ratios or ratios of medians.

All statistical tests were two-sided, and a *p*-value < 0.05 was considered statistically significant. No formal sample size calculation was performed because of the retrospective design of the study.

## 3. Results

### 3.1. Patient Characteristics

**Figure 1:**
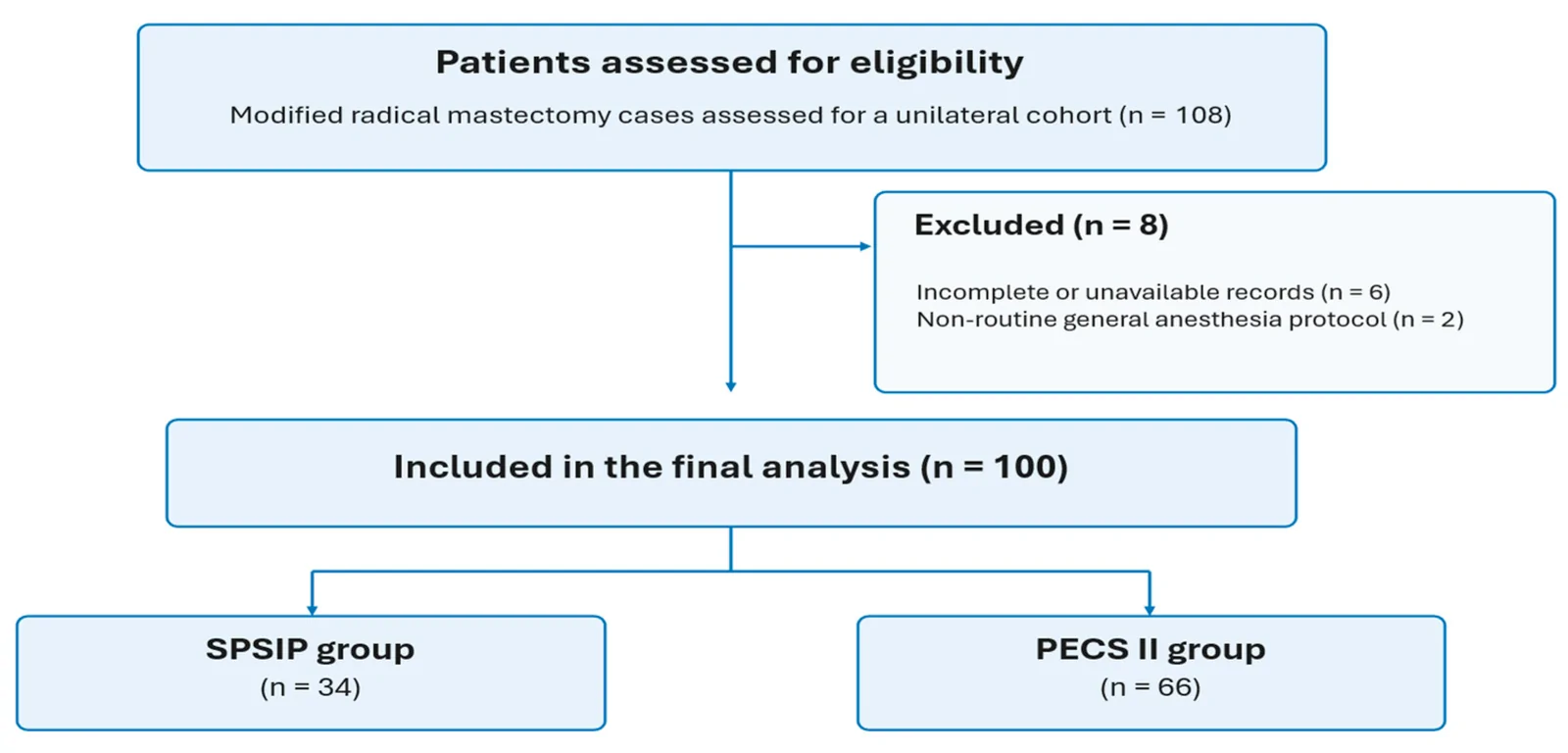
Patient selection flow diagram.

A total of 108 patients undergoing unilateral modified radical mastectomy were assessed for eligibility. Eight patients were excluded, six because of incomplete or unavailable medical records and two because anesthesia management was performed outside the routine institutional general anesthesia protocol. The remaining 100 patients were included in the final analysis, of whom 34 received an SPSIP block, and 66 received a PECS II block (Figure 1).

Data for the primary outcome, time to first rescue analgesia, were complete for all participants. One patient had missing age data and was therefore excluded from the fully adjusted multivariable model, resulting in an adjusted sample of 99 patients.

Baseline demographic and perioperative characteristics are presented in Table 1. The two groups were comparable with respect to age, body mass index (BMI), American Society of Anesthesiologists (ASA) physical status, and smoking status. However, operative duration was significantly longer in the PECS II group than in the SPSIP group (160 (126.3–200) vs. 125 (110–140) min, *p* < 0.001).

### 3.2. Primary Outcome

**Figure 2:**
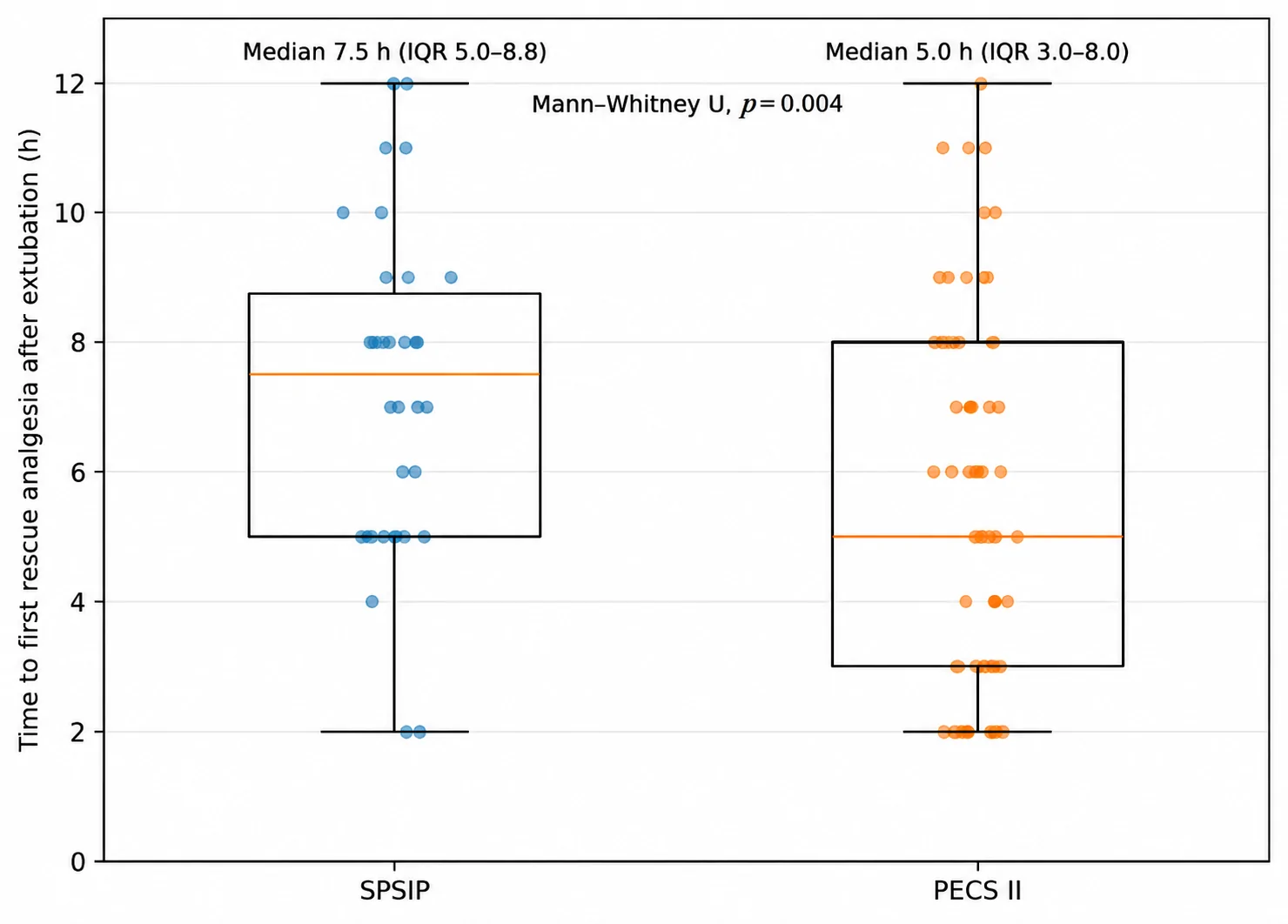
Distribution of the time to first rescue analgesia in the SPSIP and PECS II groups. Individual data points represent individual patients, and box-and-whisker plots indicate the median and interquartile range (IQR). Blue and orange dots represent individual patient observations in the SPSIP and PECS II groups, respectively. Boxes indicate the interquartile range (IQR), horizontal lines within the boxes represent the median, and whiskers extend to the minimum and maximum observed values. Between-group differences were assessed using the Mann–Whitney U test (*p* = 0.004).

The median time from extubation to the first administration of rescue analgesia was significantly longer in the SPSIP group than in the PECS II group (7.5 (5.0–8.8) vs. 5.0 (3.0–8.0) h; *p* = 0.004) (Table 2; Figure 2). All 100 patients received rescue analgesia within 12 h after extubation; therefore, the primary outcome was fully observed in all participants, with no censored observations. However, after adjustment for operative duration using a generalized linear model, this association was no longer statistically significant (exp(B) = 1.21, 95% confidence interval (CI) 0.98–1.49; *p* = 0.077). The gamma-log model demonstrated acceptable goodness of fit, with a scaled deviance-to-degrees-of-freedom ratio of 1.11 and a scaled Pearson chi-square-to-degrees-of-freedom ratio of 1.00.

Because the study was retrospective, the sample size was determined by the number of eligible patients available during the study period rather than by an a priori power calculation. Accordingly, the precision of the adjusted estimates may be limited by the sample size.

Similar findings were observed in the sensitivity analysis after additional adjustment for age, body mass index (BMI), American Society of Anesthesiologists (ASA) physical status, and smoking status (exp(B) = 1.17, 95% CI 0.94–1.46; *p* = 0.153).

### 3.3. Secondary Outcomes

Postoperative pain scores differed between the study groups at selected assessment time points. Patients who received the SPSIP block reported significantly lower Numeric Rating Scale (NRS) pain scores at 1 h after surgery than those who received PECS II (*p* < 0.001). No significant between-group difference was observed at 4 h (*p* = 0.483). In contrast, patients in the PECS II group demonstrated lower pain scores at 8 h and 12 h postoperatively (Holm-adjusted *p* = 0.013 and *p* < 0.001, respectively). Maximum postoperative NRS scores and cumulative tramadol consumption during the first 24 postoperative hours were also comparable between the groups (Table 3). Effect-size estimates based on rank-biserial correlation, together with their corresponding 95% confidence intervals, are presented in Table 3.

Cumulative opioid consumption during the first 24 postoperative hours was comparable between the SPSIP and PECS II groups (75 (50–100) vs. 75 (50–100) mg; *p* = 0.922) (Table 3).

**Figure 3:**
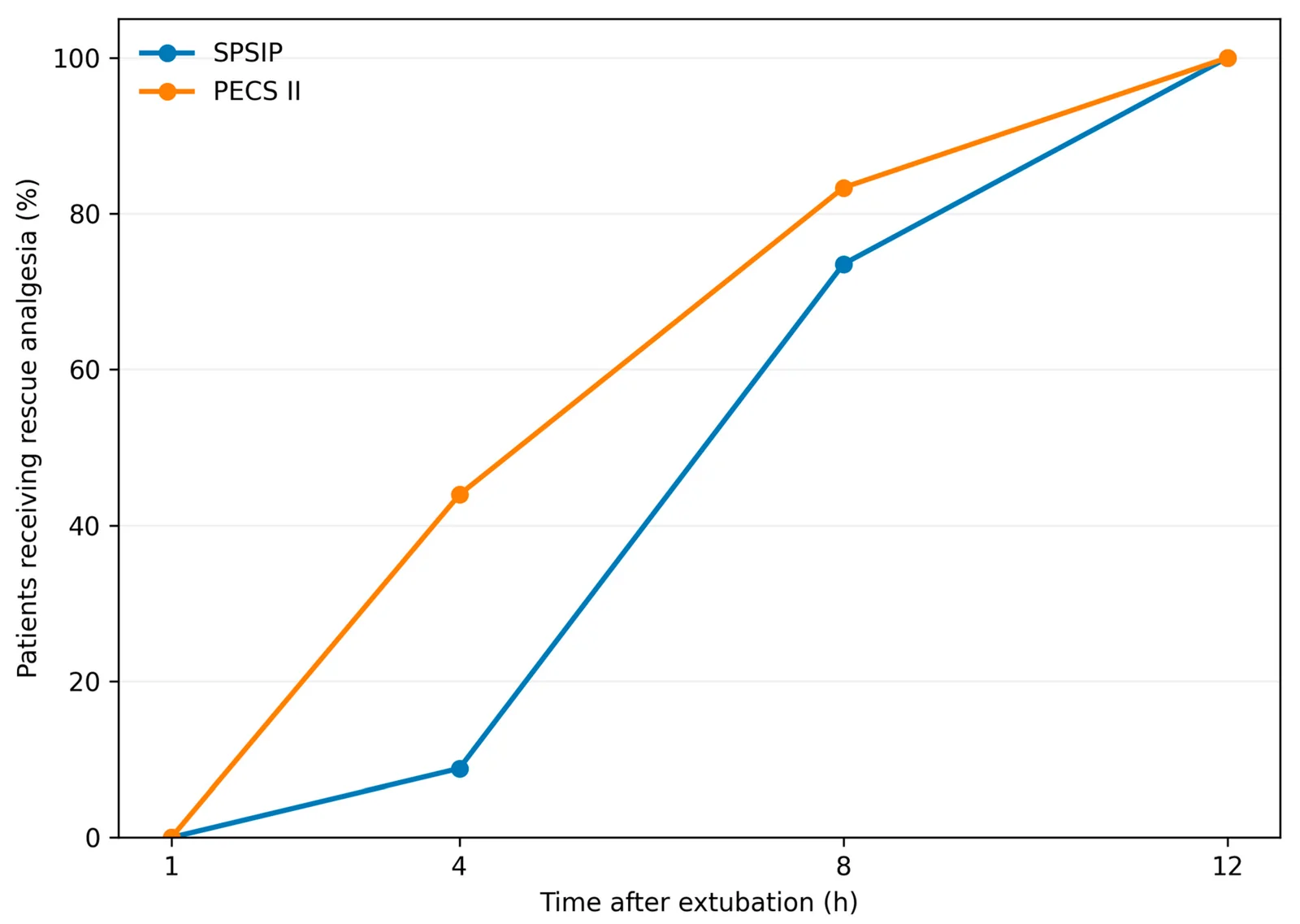
Cumulative proportion of patients who received rescue analgesia at predefined postoperative time points in the SPSIP and PECS II groups. A greater proportion of patients in the PECS II group required rescue analgesia during the early postoperative period, particularly within the first 4 h after extubation.

The cumulative distribution of rescue analgesia differed between the two groups. Within the first four postoperative hours, rescue analgesia had been administered to 3 of 34 patients (8.8%) in the SPSIP group compared with 29 of 66 patients (43.9%) in the PECS II group (Figure 3).

No documented block-related complications, including hematoma, pneumothorax, or local anesthetic systemic toxicity, were identified in the available medical records in either group.

### 3.4. Postoperative Pain Scores

Postoperative pain intensity, assessed using the Numeric Rating Scale (NRS), demonstrated different temporal patterns between the two groups.

At postoperative hour 1, NRS scores were significantly lower in the SPSIP group than in the PECS II group (2.0 (1.0–2.0) vs. 3.0 (2.0–4.8), Holm-adjusted *p* < 0.001). No significant between-group difference was observed at postoperative hour 4 (Holm-adjusted *p* = *0.483*).

In contrast, NRS scores at postoperative hours 8 and 12 were significantly lower in the PECS II group (Holm-adjusted *p* = 0.013 and *p* < 0.001, respectively).

Maximum postoperative NRS scores were similar between the groups (*p* = 0.452).

**Figure 4:**
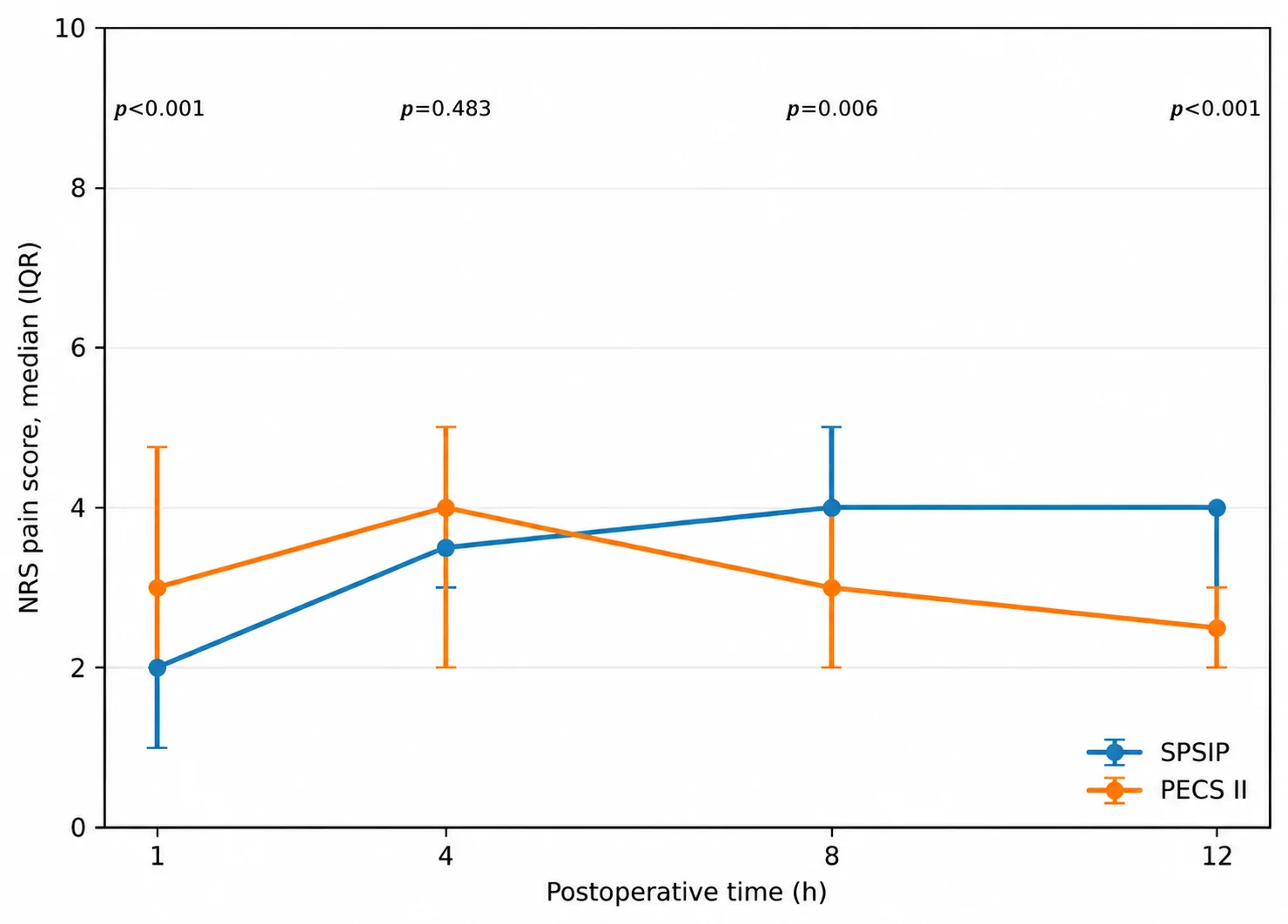
Postoperative Numeric Rating Scale (NRS) pain scores in the SPSIP and PECS II groups at 1, 4, 8, and 12 h after surgery. Points represent median values, and error bars indicate the interquartile range (IQR). *p*-values for between-group comparisons at the four predefined time points were adjusted using the Holm procedure.

The longitudinal changes in postoperative pain scores are illustrated in Figure 4.

## 4. Discussion

The present retrospective cohort study compared the postoperative analgesic efficacy of the serratus posterior superior intercostal plane (SPSIP) block and pectoral nerve block II (PECS II) following modified radical mastectomy. Although the median time to first rescue analgesia was longer in the SPSIP group than in the PECS II group, this association was no longer statistically significant after adjustment for operative duration. Similar findings were observed in the sensitivity analysis after additional adjustment for age, body mass index (BMI), ASA physical status, and smoking status. These findings do not provide sufficient evidence of an independent association between block type and time to first rescue analgesia after adjustment for the measured covariates. Importantly, the absence of statistical significance should not be interpreted as evidence of equivalence or non-inferiority between the two techniques, particularly given the confidence intervals and the limited sample size.

The analgesic efficacy of PECS II in breast surgery has been well established by randomized controlled trials and meta-analyses. Versyck et al. [2] demonstrated that PECS II significantly reduced postoperative opioid consumption and pain scores while prolonging the time to first analgesic request compared with systemic analgesia. Nevertheless, the reported duration of analgesia varies considerably across studies because of differences in surgical procedures, local anesthetic agents, adjuvant use, block timing, and criteria for rescue analgesia. Therefore, the median duration of 5 h analgesia observed in the PECS II group in the present study should be interpreted within the context of these methodological differences.

Clinical evidence regarding the use of the SPSIP block in breast surgery is relatively recent. Following the initial case reports describing the technique, randomized controlled trials have suggested that the SPSIP block may provide postoperative analgesia and reduce the need for rescue analgesia [6,7,8,9,10,11]. In our study, although the time to first rescue analgesia was longer in the SPSIP group, the association lost statistical significance after adjustment for operative duration, and similar results were obtained in the sensitivity analysis. Therefore, the initially observed difference in time to first rescue analgesia cannot be attributed solely to the independent analgesic effect of the SPSIP block.

The timing of rescue analgesia should be considered when interpreting postoperative pain scores. By postoperative hour 4, rescue analgesia had already been administered to 29 of 66 patients (43.9%) in the PECS II group compared with 3 of 34 patients (8.8%) in the SPSIP group. Although the between-group differences in NRS scores at 1, 8, and 12 h remained statistically significant after Holm adjustment for multiple comparisons, the NRS scores recorded at 8 and 12 h were obtained after a substantially greater proportion of patients in the PECS II group had already received rescue analgesia.

Consequently, the lower NRS scores observed in the PECS II group at postoperative hours 8 and 12 should be interpreted cautiously, as they may reflect the effects of additional analgesic treatment rather than the sustained analgesic efficacy of the block itself. Late postoperative NRS scores may not be suitable for direct comparisons of the duration of analgesic effect between the two block techniques. In this context, the time to first rescue analgesia represents a more appropriate outcome measure for assessing early postoperative analgesic efficacy because it is not directly affected by subsequent administrations of rescue analgesia.

Twenty-four-hour cumulative tramadol consumption was comparable between the two groups. Although the SPSIP group had a longer time to first rescue analgesia in the unadjusted analysis, this difference did not translate into reduced overall opioid consumption. These findings suggest that both the duration of analgesia and cumulative rescue analgesic requirements should be considered when evaluating the clinical effectiveness of regional analgesic techniques.

The time to first rescue analgesia after PECS II varies across published studies because of differences in block timing and technique, local anesthetic agents, adjuvant use, surgical procedures, and rescue analgesia protocols [12,13,14,15]. Among these varied findings, Mohsin et al. [13] reported a mean time of 6.53 ± 2.78 h after modified radical mastectomy, within a similar timeframe to the median of 5.0 h observed in our PECS II group.

Several regional analgesic techniques have been compared for postoperative pain management following breast surgery. Randomized studies comparing PECS II with the serratus anterior plane block have generally demonstrated effective analgesia with both techniques, with some reporting a longer duration of analgesia in favor of the serratus anterior plane block [16,17]. Likewise, recent network meta-analyses comparing PECS II with paravertebral, erector spinae plane, and serratus plane blocks have reported heterogeneous findings [18,19]. As novel interfascial plane blocks continue to emerge, comparative clinical studies remain essential to clarify their relative efficacy and define their role in routine clinical practice. The present study contributes to this growing body of evidence by providing one of the first direct clinical comparisons between SPSIP and PECS II in patients undergoing modified radical mastectomy.

### Limitations

This study has several limitations. First, the retrospective, single-center design and the absence of randomization introduce the possibility of selection bias in block allocation and residual confounding. Although statistical adjustment helps account for measured confounding factors, residual confounding cannot be excluded. Covariates were selected based on their clinical relevance and potential associations with postoperative outcomes; however, the available data may not have permitted comprehensive adjustment for all relevant confounding factors. Detailed information on the extent of axillary dissection, surgical complexity, surgeon and block-performing anesthesiologist, prior opioid exposure, and calendar-period effects was not captured in the study dataset and therefore could not be incorporated into the adjusted analyses. Consequently, residual confounding related to these unmeasured factors cannot be excluded. Although perioperative analgesic management followed a standardized institutional protocol, cumulative intraoperative remifentanil exposure and other detailed perioperative management variables were not consistently available in the retrospective dataset. Therefore, residual confounding related to differences in intraoperative opioid exposure or perioperative management cannot be excluded.

Second, the study groups were unequal in size (SPSIP, *n* = 34; PECS II, *n* = 66), reflecting the retrospective nature of the study. Although unequal group sizes do not necessarily invalidate a comparison, the smaller SPSIP group may have resulted in less precise estimates and limited statistical power. Although the robustness of the findings was assessed using a primary model adjusted for operative duration and a sensitivity analysis incorporating additional clinical covariates, the limitations inherent to the retrospective design and the possibility of residual confounding cannot be entirely excluded.

Third, a standardized sensory assessment of block success and dermatomal spread was not systematically documented in the medical records. Therefore, incomplete or failed blocks could not be reliably identified, and the proportion of patients with confirmed successful sensory blockade could not be determined. Accordingly, the present findings reflect the clinical effectiveness of SPSIP and PECS II as performed in routine practice rather than the efficacy of sensory-confirmed successful blocks.

Although NRS scores were recorded before rescue analgesia was administered at each assessment, NRS scores at later postoperative time points may have been influenced by rescue analgesia administered previously. Therefore, NRS scores recorded at postoperative hours 4, 8, and 12 should not be interpreted as independent measures of block efficacy.

Finally, postoperative pain scores were available only during the first 12 postoperative hours because of missing data, limiting the assessment of longer-term postoperative pain trajectories.

## 5. Conclusions

In this retrospective study, the time to first rescue analgesia was longer in the SPSIP group in the unadjusted analysis; however, this association was no longer statistically significant after adjustment for operative duration, and the sensitivity analysis yielded similar findings. Postoperative NRS scores showed different temporal patterns between the two groups, while 24-h tramadol consumption was comparable. Because rescue analgesia was administered earlier and more frequently in the PECS II group, later postoperative pain scores should be interpreted cautiously. Taken together, these findings suggest that SPSIP may provide effective early postoperative analgesia and may represent a clinically feasible regional analgesic option for patients undergoing modified radical mastectomy.

## Figures and Tables

**Table 1:** Baseline Demographic and Perioperative Characteristics.

Characteristic	SPSIP (*n* = 34)	PECS II (*n* = 66)	*p*-Value
Age, years	56 (50–65)	54 (48–61)	0.195
Body mass index, kg/m^2^	25.7 (22.9–29.1)	26.0 (24.0–31.0)	0.560
ASA physical status, *n* (%)	I: 2 (5.9) II: 26 (76.5) III: 6 (17.6)	I: 13 (19.7) II: 41 (62.1) III: 12 (18.2)	0.170
Current smokers, *n* (%)	17 (50.0)	27 (40.9)	0.386
Operative duration, min	125 (110–140)	160 (126.3–200)	<0.001

Values are presented as median (interquartile range) unless otherwise indicated. Continuous variables were compared using the Mann–Whitney U test, and categorical variables using Pearson’s chi-square test or Fisher’s exact test, as appropriate. Abbreviations: ASA, American Society of Anesthesiologists; IQR, interquartile range.

**Table 2:** Primary Outcome Analysis.

Outcome	SPSIP (*n* = 34)	PECS II (*n* = 66)	Adjusted Effect Estimate (exp(B))	95% CI	*p*-Value
Time to first rescue analgesia, h (unadjusted)	7.5 (5.0–8.8)	5.0 (3.0–8.0)	—	—	0.004
Adjusted GENLIN model *	—	—	1.21	0.98–1.49	0.077
Sensitivity analysis †	—	—	1.17	0.94–1.46	0.153

Values are presented as median (interquartile range) unless otherwise indicated. exp(B) represents the ratio of model-based expected mean times to first rescue analgesia and should not be interpreted as a hazard ratio or a ratio of medians. * Generalized linear model with gamma distribution and log-link function adjusted for operative duration. † Additionally adjusted for operative duration, age, body mass index, ASA physical status, and smoking status. Abbreviations: CI, confidence interval; GENLIN, generalized linear model.

**Table 3:** Secondary Postoperative Outcomes.

Outcome	SPSIP (*n* = 34)	PECS II (*n* = 66)	Effect Size, rᵣᵦ (95% CI)	*p*-Value
NRS score at 1 h	2.0 (1.0–2.0)	3.0 (2.0–4.8)	−0.52 (−0.70 to −0.33)	<0.001 *
NRS score at 4 h	3.5 (3.0–5.0)	4.0 (2.0–5.0)	0.08 (−0.13 to 0.30)	0.483 *
NRS score at 8 h	4.0 (3.0–5.0)	3.0 (2.0–4.0)	0.33 (0.11 to 0.54)	0.013 *
NRS score at 12 h	4.0 (3.0–4.0)	2.5 (2.0–3.0)	0.52 (0.31 to 0.71)	<0.001 *
Maximum NRS score	6.0 (5.0–7.0)	6.0 (5.0–6.8)	0.09 (−0.14 to 0.32)	0.452
24-h tramadol consumption, mg	75 (50–100)	75 (50–100)	0.01 (−0.20 to 0.22)	0.922

Values are presented as median (interquartile range). Rank-biserial correlation (rᵣᵦ) is reported as the effect-size estimate with corresponding 95% confidence intervals. Negative values indicate lower values in the SPSIP group, whereas positive values indicate higher values in the SPSIP group. * *p*-values for between-group comparisons of NRS scores at 1, 4, 8, and 12 h were adjusted for multiple comparisons using the Holm procedure. Abbreviations: CI, confidence interval; NRS, Numeric Rating Scale.

## Data Availability

The datasets generated and/or analyzed during the current study are available from the corresponding author upon reasonable request.

## Abbreviations

The following abbreviations are used in this manuscript:

SPSIP	Serratus Posterior Superior Intercostal Plane
PECS II	Pectoral Nerve Block II
IQR	Interquartile Range
ASA	American Society of Anesthesiologists
BMI	Body Mass Index
